# Autophagy promotes apoptosis of mesenchymal stem cells under inflammatory microenvironment

**DOI:** 10.1186/s13287-015-0245-4

**Published:** 2015-12-15

**Authors:** Shipeng Dang, Zhi-ming Yu, Chang-ying Zhang, Jie Zheng, Ku-lin Li, Ying Wu, Ling-ling Qian, Zhen-yu Yang, Xiao-rong Li, Yanyun Zhang, Ru-xing Wang

**Affiliations:** Department of Cardiology, Wuxi People’s Hospital Affiliated to Nanjing Medical University, Wuxi, 214023 China; Key Laboratory of Stem Cell Biology, Institute of Health Sciences, Shanghai Institutes for Biological Sciences, Chinese Academy of Sciences and Shanghai Jiao Tong University School of Medicine (SJTUSM), Shanghai, 200025 China; Shanghai Institute of Immunology, Institutes of Medical Sciences, Shanghai Jiao Tong University School of Medicine (SJTUSM), Shanghai, 200025 China

**Keywords:** Mesenchymal stem cells, Autophagy, Apoptosis, Sepsis, Bcl-2

## Abstract

**Background:**

Mesenchymal stem cells (MSCs) have been widely applied to treat various inflammatory diseases. Inflammatory cytokines can induce both apoptosis and autophagy in MSCs. However, whether autophagy plays a pro- or con-apoptosis effect on MSCs in an inflammatory microenvironment has not been clarified.

**Methods:**

We inhibited autophagy by constructing MSCs with lentivirus containing small hairpin RNA to knockdown *Beclin-1* and applied these MSCs to a model of sepsis to evaluate therapeutic effect of MSCs.

**Results:**

Here we show that inhibition of autophagy in MSCs increases the survival rate of septic mice more than control MSCs, and autophagy promotes apoptosis of MSCs during application to septic mice. Further study demonstrated that autophagy aggravated tumor necrosis factor alpha plus interferon gamma-induced apoptosis of MSCs. Mechanically, autophagy inhibits the expression of the pro-survival gene *Bcl-2* via suppressing reactive oxygen species/mitogen-activated protein kinase 1/3 pathway.

**Conclusions:**

Our findings indicate that an inflammatory microenvironment-induced autophagy promotes apoptosis of MSCs. Therefore, modulation of autophagy in MSCs would provide a novel approach to improve MSC survival during immunotherapy.

## Background

Mesenchymal stem cells (MSCs) are a heterogeneous population of multipotent stromal stem cells that can be obtained from many tissues [1]. They not only possess self-renew capability, but also they can differentiate into a variety of mesodermal lineage cells, such as adipocytes, osteocytes, chondrocytes, cadiomyocytes, and so forth [2, 3]. In addition, MSCs play an important modulatory role in both the innate and adaptive immune systems through interaction with the inflammatory microenvironment [4, 5]. Therefore, MSCs have engaged great interest for clinical applications in treating various inflammatory diseases, such as acute graft-versus-host disease [6], multiple sclerosis [7], rheumatoid arthritis [8], bone injury and sepsis [9, 10]. However, there is little insight into the specific role of the inflammatory microenvironment on the fate of MSCs.

The inflammatory microenvironment in vivo is crucial for MSCs to mediate their immunoregulatory capability [5, 11]. Inflammatory cytokines such as interferon (IFN)-γ, tumor necrosis factor (TNF)-α, interleukin (IL)-1α and IL-β, can activate MSCs to exert immunosuppressive functions [3, 12]. Previous studies have shown that IL-2-activated natural killer cells could efficiently lyse autologous and allogeneic MSCs [13]. In addition, it has been reported that IFN-γ synergistically enhances TNF-α-induced MSC apoptosis, which inhibits the ability of MSCs to mediate bone repair [9], suggesting that the inflammatory microenvironment induced apoptosis of MSCs in treating inflammatory diseases. Our previous study has demonstrated that inflammatory cytokines, e.g., IFN-γ and TNF-α, induce autophagy in MSCs synergistically by inducing expression of Beclin 1, whereas autophagy, in return, downregulates the immunosuppressive function of MSCs. Furthermore, inhibition of autophagy by knockdown of Beclin 1 significantly improves the therapeutic effects of MSCs on experimental autoimmune encephalomyelitis (EAE) [14]. However, whether inflammation-induced autophagy plays a role in regulating MSC apoptosis remains elusive.

Sepsis is a serious medical condition, characterized by a generalized inflammatory state caused by infection [15]. As a model of sepsis, the process of cecal ligation and puncture (CLP) closely resembles the human disease [16, 17]. It has been reported that MSCs attenuate sepsis via by acting on the monocytes, macrophages or neutrophils in the CLP mouse model [10, 18–20]. In this study, we show that inhibition of autophagy in MSCs increases the survival rate of septic mice more than control MSCs, using the CLP model. This elevation is mainly due to autophagy-aggravated TNF-α plus IFN-γ-induced apoptosis of MSCs. Our findings identify autophagy to be a critical regulator of the apoptosis of MSCs during their application for inflammatory diseases.

## Materials and methods

### MSC isolation and culture

Bone marrow MSCs were isolated as previously described [14]. Briefly, bone marrow cells were flushed out from the bone cavity of femurs and tibias of mice with Dulbecco’s modified Eagle's medium low glucose (L-DMEM, Hyclone, USA) containing 10 % fetal bovine serum (FBS; Gibco, USA). A single-cell suspension of all nucleated cells was obtained by passing all bone marrow cells through a 70-μm cell strainer (BD Bioscience, USA). All the single cells were seeded at 1 × 10^6^ into 100-mm culture dishes (Corning, USA) and initially incubated for 48 hours at 37 °C and 5 % CO_2_. To eliminate the nonadherent cells, the cultures were washed with phosphate-buffered saline (PBS; Gibco) twice on the second day. The attached cells were cultured with L-DMEM supplemented with 15 % FBS, 2 mM L-glutamine (Invitrogen, USA), 100 U/ml penicillin and 100 μg/ml streptomycin (Invitrogen). Cell surface marker analysis confirmed that MSCs were positive for CD44 and Sca-1, and negative for CD11b, CD31, CD34, CD45, major histocompatibility complex (MHC) class I and MHC class II (eBioscience, USA).

### Lentiviral vector construction

Oligonucleotides with the following nucleotide sequences were used for the cloning of small hairpin (sh)RNA-encoding sequences into a lentiviral vector: *Beclin-1*: 5′-gatccGGAGAAAGGCAAGATTGAAGATTCAAGAGATCTTCAATCTTGCCTTTCTCCTTTTTTg-3′; negative controls (NC): 5′-gatccACTACCGTTGTTATAGGTGTTCAAGAGACACCTATAACAACGGTAGTTTTTTTg-3′. High-titer lentiviral stocks were produced and used at a multiplicity of infection of 50 to infect MSCs and the efficiency of infection exceeded 95 %. MSCs were stably infected with negative control lentivirus (shNC-MSCs) or lentivirus expressing shRNA inhibiting the essential autophagy gene *Beclin-1* (shBec1-MSCs).

### Immunoblot

Cells were lysed with ice-cold RIPA containing protease and phosphatase inhibitors (Roche, Switzerland). The lysates were fractionated by SDS-PAGE and analyzed by immunoblotting with specific antibodies to LC3 I/II, GAPDH, Beclin 1, Bcl-2, and cleaved Caspase 3 (Cell Signaling, USA).

### CLP model induction and treatment

CLP was performed as previously described in C57BL/6 J mice [10, 21]. Briefly, the mice were anesthetized and the cecum was ligated by silk 4-0 and punctured twice with a 21-gauge needle, squeezed gently to express a small amount of fecal material and then returned to the central abdominal cavity. In sham-operated mice, the cecum was located but neither ligated nor punctured. The abdominal incision was closed in two layers with 6-0 nylon sutures. After surgery, all mice received antibiotic and fluid therapy subcutaneously. PBS, shNC-MSCs or shBecn1-MSCs (1 × 10^6^ cells/mouse) were administered intravenously after the surgery. Survival rate was assessed after surgery every 8 hours. All aspects of the animal care and experimental procedures were in accordance with the Guide for the Care and Use of Laboratory Animals and approved by the Experimental Animal Care and Use Committee of Nanjing Medical University.

### Apoptosis in vivo

Apoptosis assay in vivo was performed as previously described [9]. Briefly, shNC-MSCs and shBec1-MSCs were suspended in PBS with 2 % FBS, mixed with high concentration matrigel matrix (BD Biosciences), and then injected on the back of naive and sepsis mice subcutaneously for 24 hours. shNC-MSCs and shBec1-MSCs were then isolated and stained with annexin V/prodium iodide (PI) apoptosis detection kit (Invitrogen) and measured by flow cytometry (BD Aria, USA).

### Apoptosis induction with TNF-α and IFN-γ

shNC-MSCs and shBec1-MSCs were stimulated with TNF-α (20 ng/ml; R&D Systems, USA) plus IFN-γ (50 ng/ml; R&D Systems) for 24 hours. In some experiments, mitogen-activated protein kinase 1/3 (ERK) inhibitor (PD98059; Merck, Germany) and reactive oxygen species (ROS) inhibitor (N-acetyl cysteine (NAC); Sigma-Aldrich) were added. In some other experiments, naive MSCs were treated with 3-methyladenine (3-Ma; 10 mmol/L; Sigma-Aldrich) for 12 hours before stimulation with TNF-α plus IFN-γ. Then cells were stained with annexin V/PI and measured by flow cytometry.

### Cytokine detection

The sera of naive and sepsis mice were quantified with TNF-α, IFN-γ, IL-6 and IL-17 enzyme-linked immunosorbent assay (ELISA) kit according to the manufacturer’s guidelines (R&D Systems).

### Quantitative real-time polymerase chain reaction

Total RNA was extracted from MSCs at the indicated times and was subsequently reverse-transcribed using a Reverse Transcription System (Takara, Japan). Quantitative polymerase chain reaction (PCR) was performed using SYBR Green PCR mix (Roche) on an ABI Prism® 7900HT Sequence Detection System (Applied Biosystems). β-actin was used as an internal control to normalize for differences in the amount of total RNA in each sample. The primer sequences are listed as follows (in the 5 to 3 orientation): *Bcl-2* forward GGGCATAGTGAAGGCAGGAA, reverse TCCATAGACACGGGTCATCGA; *Bcl-xl* forward AGGTATTGGTGAGTCGGATTG, reverse TCTCGGCTGCTGCATTGTT; *Bim* forward CTACATGCAGCCAGGATACGT; reverse ACACTGTCGCAGCACCAAGA; *Mcl-1* forward TTTCTTTCGGTGCCTTTGTGG, reverse CAGTCCCGTTTCGTCCTTACA.

### Statistics

SPSS 17.0 was used to perform the statistical analysis. Significance was assessed using an independent two-tailed Student’s *t* test or with analysis of variance. We compared survival curves with a log-rank test (Graphpad Prism 5.0). *P* < 0.05 was considered statistically significant.

## Results

### Autophagy promotes apoptosis of MSCs under sepsis state

**Fig. 1:**
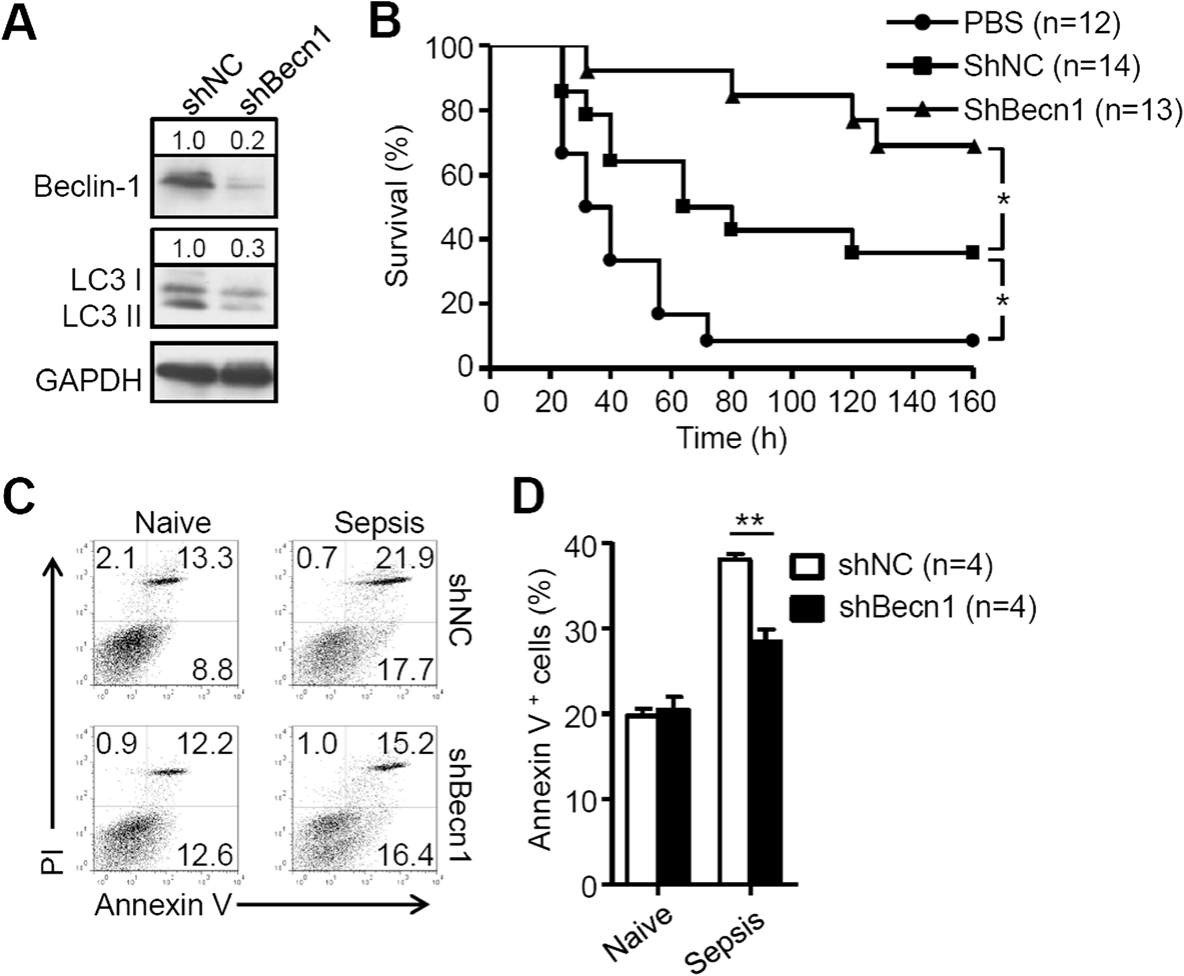
Inhibition of autophagy improves survival of MSCs in CLP mice. **a** MSCs were stably infected with negative control lentiviral vector (*shNC*) or with vector expressing shRNA to inhibit *Beclin-1* (*shBecn1*); western blot analysis of shNC-MSCs and shBec1-MSCs. **b** Survival curves of CLP mice treated with phosphate-buffered saline (*PBS*; n = 12 mice per group), shNC-MSCs (n = 14 mice per group) or shBec1-MSCs (n = 13 mice per group) intravenously (1 × 10^6^ cells/mouse). **c**, **d** shNC-MSCs or shBec1-MSCs were mixed with matrigel and were injected into naive and CLP mice subcutaneously for 24 hours. **c** Then MSCs were isolated and stained with annexin V/prodium iodide (*PI*) for apoptosis assay. **d** Percentage of MSC apoptosis after injection into naive (n = 4 mice per group) and CLP mice (n = 4 mice per group); data are shown as mean ± SEM. *P < 0.05, **P < 0.01

It has been reported that inflammatory cytokines in bone injury, mainly TNF-α and IFN-γ, are capable of inducing MSC apoptosis [9]. Our previous study showed that the inflammatory microenvironment could induce autophagy of MSCs [14]. To investigate whether inflammatory cytokine-induced autophagy regulated apoptosis of MSCs, we inhibited autophagy by constructed MSCs with lentivirus containing shRNA to knockdown *Beclin-1*, a key regulator of autophagy. As shown in Fig. 1a, the expression of Beclin-1 was markedly decreased on immunoblots in shBec1-MSCs in parallel with reduced level of LC3-II, indicating that knockdown of *Beclin-1* was effective in suppressing autophagy. Then we assessed survival rates of CLP mice treated with PBS, shNC-MSCs or shBec1-MSCs. There was a significant (*P* < 0.05) improvement in the survival of mice treated with shBec1-MSCs compared to shNC-MSCs (Fig. 1b). Next, to explore whether reduction of apoptosis played a role in the elevated survival rate of shBec1-MSC-treated mice, shNC-MSCs or shBec1-MSCs were mixed with matrigel and injected subcutaneously into naive and CLP mice. Our data demonstrated that the percentage of apoptotic shBec1-MSCs and shNC-MSCs were 28.5 ± 1.4 % and 38.0 ± 0.7 %, respectively (*P* < 0.01) (Fig. 1c, d). These results suggest that autophagy enhances apoptosis of MSCs in the inflammatory microenvironment, which may result in reduction of the therapeutic effect of MSCs on CLP.

### Autophagy promotes the apoptosis of MSCs induced by inflammatory cytokines

**Fig. 2:**
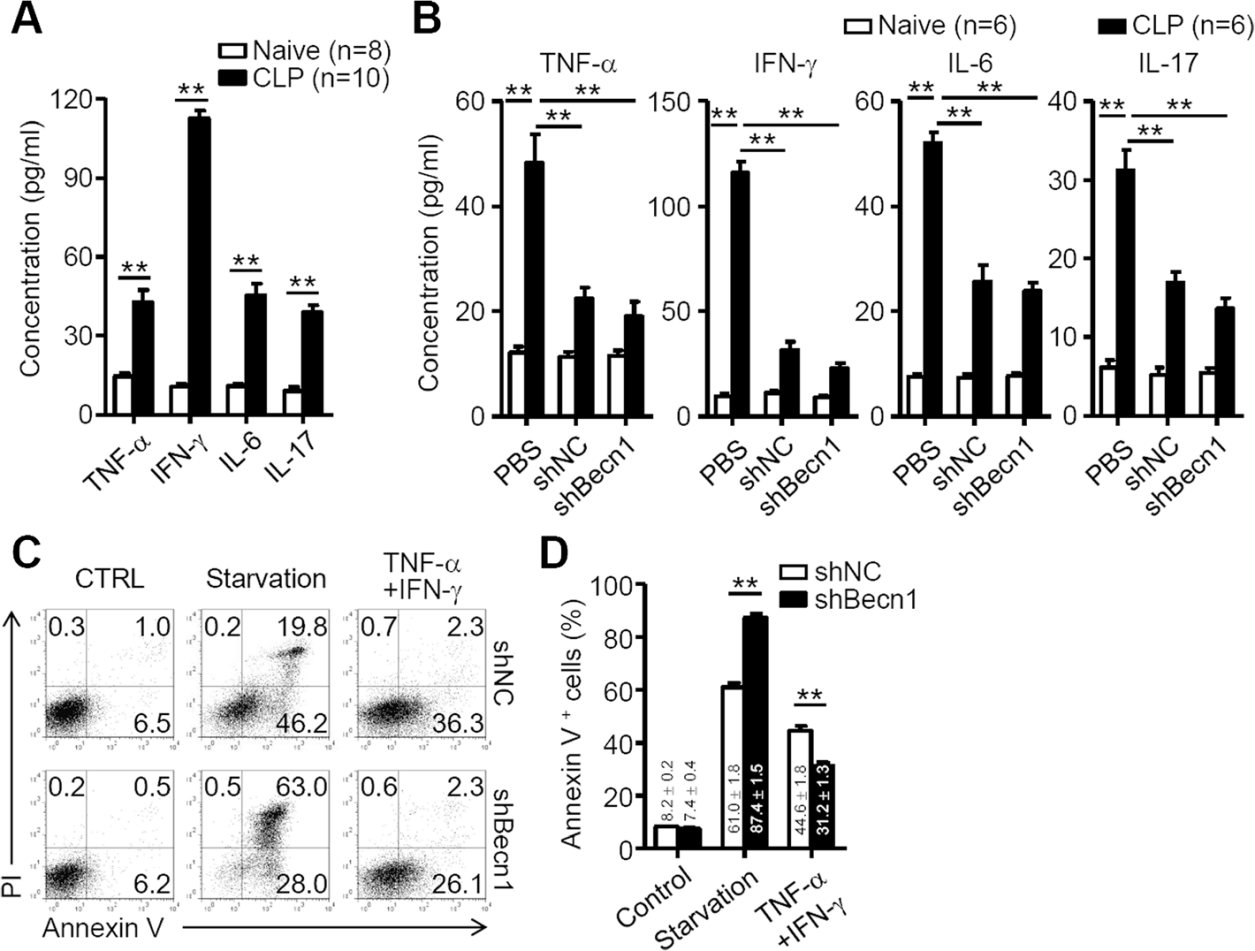
Inhibition of autophagy moderately suppresses tumor necrosis factor alpha (*TNF-α*) plus interferon gamma (*IFN-γ*)-induced apoptosis of MSCs. **a** Levels of cytokines in sera of naive mice (n = 8 mice per group) and cecal ligation and puncture (*CLP*) mice (n = 10 mice per group) were determined by ELISA. Data are shown as mean ± SEM. **b** Naive or CLP mice were treated with phosphate-buffered saline (*PBS*; n = 6 mice per group), shNC-MSCs (*shNC*; n = 6 mice per group) or shBec1-MSCs (*shBecn1*; n = 6 mice per group) intravenously (1 × 10^6^ cells/mouse) for 8 hours after the surgery. Levels of cytokines in sera of naive mice and CLP mice were determined by ELISA. Data are shown as mean ± SEM. **c** shNC-MSCs and shBec1-MSCs were stimulated with TNF-α (20 ng/ml) plus IFN-γ (50 ng/ml) or starved with EBSS for 24 hours, and then the cells were harvested and stained with annexin V/prodium iodide (*PI*). **d** Percentage of MSC apoptosis after treatment with TNF-α plus IFN-γ; data are shown as mean ± SEM of five independent experiments. **P < 0.01. *CTRL* Control

To investigate how autophagy enhances apoptosis of MSCs in the inflammatory microenvironment, we assessed the inflammatory cytokines TNF-α, IFN-γ, IL-6 and IL-17 in CLP mice. Our data demonstrated that there was a significant elevation of these cytokines in CLP mice compared to naive mice (Fig. 2a). Then we investigated the expression of these inflammatory cytokines after treatment with shBec1-MSCs/shNC-MSCs. As shown in Fig. 2b, treatment with shBec1-MSCs/shNC-MSCs significantly reduced the expression of these inflammatory cytokines in CLP mice but not in naive mice, and this effect was no different between shBec1-MSCs and shNC-MSCs. Furthermore, we treated shNC-MSCs or shBecn1-MSCs with TNF-α plus IFN-γ, main cytokines that could induce apoptosis of MSCs, or starvation conditions. Firstly, we found that inhibition of autophagy promoted the apoptosis of MSCs under starvation. Then we showed that treatment with TNF-α combined with IFN-γ significantly induced apoptosis of MSCs, whereas inhibition of autophagy suppressed apoptosis of MSCs (shNC-MSCs 44.6 ± 1.7 % versus shBec1-MSCs 33.2 ± 1.5 %, *P* = 0.01) (Fig. 2c, d). These data suggest that autophagy promotes survival of MSCs under starvation but enhances apoptosis of MSCs under the inflammatory microenvironment.

### Autophagy promotes apoptosis of MSCs via downregulation of Bcl-2

**Fig. 3:**
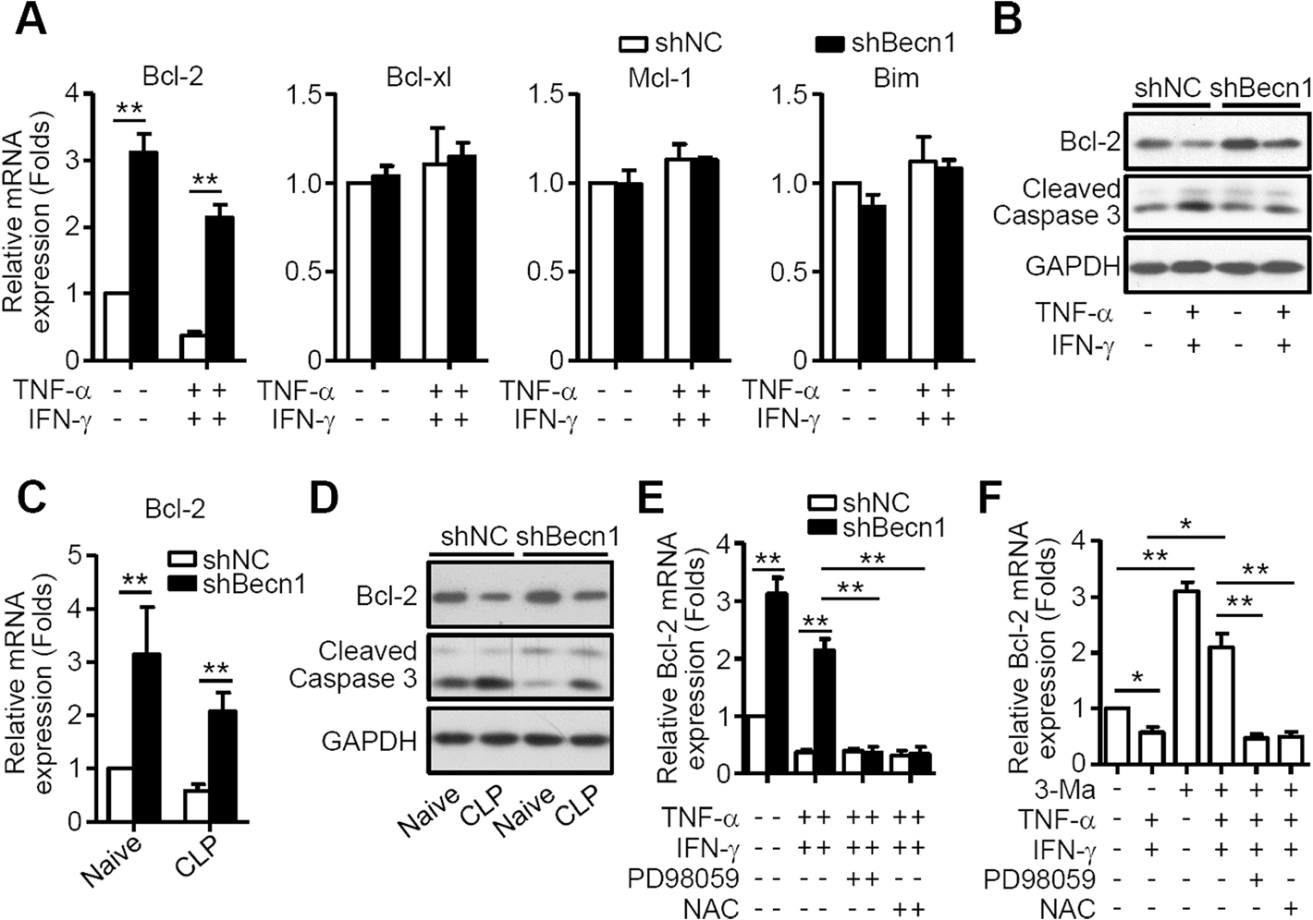
Inhibition of autophagy promotes survival of MSCs via upregulation of Bcl-2. **a** shNC-MSCs (*shNC*) and shBec1-MSCs (*shBecn1*) were stimulated with tumor necrosis factor alpha (*TNF-α*; 20 ng/ml) plus interferon gamma (*IFN-γ*; 50 ng/ml) for 4 hours. Expression of *Bcl-2*, *Bcl-xl*, *Mcl-1* and *Bim* was then measured by quantitative real-time PCR. Data are shown as mean ± SEM of four independent experiments. **b** Western blot analysis of shNC-MSCs and shBec1-MSCs stimulated with TNF-α (20 ng/ml) plus IFN-γ (50 ng/ml) for 6 hours. **c, d** shNC-MSCs or shBec1-MSCs were mixed with matrigel and were injected into naive (n = 3 mice per group) and cecal ligation and puncture (*CLP*) mice (n = 3 mice per group) subcutaneously for 24 hours. Expression of Bcl-2 was then measured by quantitative real-time PCR (**c**) and western blot (**d**). **e** shNC-MSCs and shBec1-MSCs were stimulated with TNF-α (20 ng/ml) plus IFN-γ (50 ng/ml), or with PD98059 (20 μM) or N-acetyl cysteine (*NAC*; 10 mM) for 4 hours. Expression of *Bcl-2* was then measured by quantitative real-time PCR. Data are shown as mean ± SEM of four independent experiments. **f** MSCs were pretreated with 3-methyladenine (*3-Ma*) for 12 hours, and stimulated with TNF-α (20 ng/ml) and IFN-γ (50 ng/ml), or combined with PD98059 (20 μM) or NAC (10 mM) for 4 hours. The expression of *Bcl-2* was then measured by quantitative real-time PCR. Data are shown as mean ± SEM of four independent experiments. *P < 0.05, **P < 0.01

To explore the mechanisms of autophagy-regulated MSC apoptosis, genes associated with apoptosis, e.g., *Bim*, *Bcl-2*, *Bcl-xl*, and *Mcl-1*, were examined with quantitative PCR. The results showed that TNF-α plus IFN-γ treatment inhibited the expression of the anti-apoptotic gene *Bcl-2*, and inhibition of autophagy markedly increased the constitutive mRNA levels of *Bcl-2* (Fig. 3a), suggesting that autophagy inhibited the expression of Bcl-2. Studies have shown that Bcl-2 family proteins regulate caspase activation-mediated apoptosis through controlling the release of cytochrome c [22, 23]. Our data demonstrated that autophagy inhibited the protein levels of Bcl-2 and promoted TNF-α plus IFN-γ-induced activation of caspase 3 (Fig. 3b), which enhanced TNF-α plus IFN-γ-induced apoptosis of MSCs. To determine whether a similar effect could be observed in the inflammatory environment in vivo, shNC-MSCs or shBec1-MSCs were implanted on to the back of naive and sepsis mice, and were then isolated for Bcl-2 and cleaved caspase 3 expression. Our results show that the expression of Bcl-2 was lower in CLP mice than in naive mice, and inhibition of autophagy increased the expression of Bcl-2 but decreased the expression of cleaved caspase 3 (Fig. 3c, d), results consistent with the data in vitro. We had previously demonstrated that autophagy inhibited the activation of the ROS/ERK pathway [14], and there is abundant evidence that activation of the ERK pathway can increase the expression of several pro-survival proteins, notably Bcl-2, Bcl-xl and Mcl-1, in a variety of cell types [23–25]. Therefore, we investigated activation of the ROS/ERK pathway on the expression of Bcl-2 and found that blocking activation of ERK inhibited the expression of Bcl-2 as well as inhibition of ROS (Fig. 3e). To confirm this effect resulted from inhibition of autophagy, but not from inhibition of Beclin 1, we applied the autophagy inhibitor, 3-Ma, in this experiment, and obtained the same results (Fig. 3f). These data suggest that autophagy inhibits the expression of Bcl-2 via suppressing the ROS/ERK pathway.

### Inhibition of the ROS/ERK pathway is responsible for autophagy-mediated apoptosis of MSCs under inflammatory conditions

**Fig. 4:**
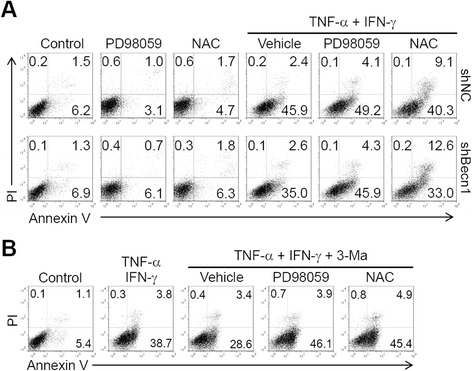
Inhibition of the ROS/ERK pathway is responsible for autophagy-mediated apoptosis of MSCs. **a** shNC-MSCs (*shNC*) and shBec1-MSCs (*shBecn1*) stimulated with tumor necrosis factor alpha (*TNF-α*; 20 ng/ml) plus interferon gamma (*IFN-γ*; 50 ng/ml) or combined with PD98059 or N-acetyl cysteine (*NAC*) before stimulation with TNF-α for 24 hours. **b** Naive MSCs were treated with 3-methyladenine (*3-Ma*; 10 mM) for 12 hours, then stimulated with TNF-α (50 ng/ml) plus IFN-γ (50 ng/ml) or combined with PD98059 or NAC before TNF-α stimulation for 24 hours. Cells were stained with annexin V/prodium iodide (*PI*) for apoptosis assay. Representative graphs from three independent experiments are shown

To investigate whether the ROS/ERK pathway was involved in autophagy-mediated apoptosis of MSCs, we treated shNC-MSCs or shBecn1-MSCs with TNF-α plus IFN-γ, and with ROS or ERK inhibitor. The data showed that inhibiting ERK activation or ROS generation considerably enhanced TNF-α plus IFN-γ-induced apoptosis of shBec1-MSCs to a level similar to that of shNC-MSCs (Fig. 4a). This result was also validated by inhibition of autophagy with 3-Ma (Fig. 4b). Collectively, these data suggest that autophagy promotes TNF-α plus IFN-γ-induced apoptosis of MSCs through inhibiting the ROS/ERK pathway-mediated anti-apoptotic function.

## Discussion

**Fig. 5:**
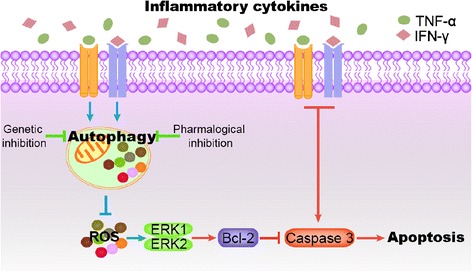
The role of autophagy in inflammatory cytokine-induced apoptosis of MSCs. When MSCs were transplanted into the inflammatory microenvironment of CLP mice, inflammatory cytokines, such as tumor necrosis factor alpha (*TNF-α*) and interferon gamma (*IFN-γ*), induced apoptosis of MSCs by activating caspase 3. Meanwhile, these inflammatory cytokines induced autophagy of MSCs. Autophagy also inhibited the expression of bcl-2 via suppression of the reactive oxygen species (ROS)/mitogen-activated protein kinase 1/3 (ERK) pathway, which promoted the activation of caspase 3 and led to apoptosis of MSCs. On the contrary, inhibition of autophagy in MSCs activated the ROS/ERK pathway, which reduced apoptosis of MSCs via upregulating Bcl-2 expression and inhibiting caspase 3 activation

Recently, MSCs have been found to be effective in treating inflammatory disease [4]. An inflammatory condition that activates MSCs is critical for MSCs to perform immunoregulation [5]. Here we demonstrated for the first time that autophagy enhanced apoptosis of MSCs under septic conditions, whereas inhibition of autophagy in MSCs increased the survival rate of septic mice. Moreover, we found that autophagy promoted TNF-α plus IFN-γ-induced apoptosis of MSCs via inhibiting the expression of Bcl-2. This effect was due to autophagy suppressing the ROS/ERK pathway (Fig. 5). Our findings suggest that modulation of autophagy in MSCs may present a novel strategy to improve MSC survival in sepsis and other inflammatory diseases.

Sepsis is a clinical syndrome occurring in patients following severe infection or injury, and is a leading cause of morbidity and mortality worldwide [15]. Recent studies have demonstrated that MSCs can alleviate the development of sepsis [20]; the effects include bacterial clearance, suppression of inflammation, anti-apoptosis, or stimulation of regenerative responses [18, 19, 26]. Németh et al. [10] report that MSCs attenuate sepsis through secreting prostaglandin E2 (PGE2). Our previous study demonstrates that inflammatory cytokines such as TNF-α and IFN-γ that are produced in EAE induce autophagy of MSCs, whereas inhibition of autophagy increases PGE2 production to augment the immunoregulatory effect of MSCs on EAE [14]. In this study, we found that inhibition of autophagy in MSCs increased the survival rate of septic mice. In addition to promoting MSC survival, this effect may be partially associated with inhibition of autophagy in MSCs upregulating PGE2 secretion. Therefore, inhibition of autophagy might be an optimal approach to improve the therapeutic effect of MSCs.

Autophagy is an evolutionarily conserved catabolic process by which cells deliver cytoplasmic components for degradation into lysosomes [27]. Despite the considerable advances in the biology of autophagy, our understanding of the dual role of autophagy in cell survival and cell death remains incomplete. It may play a pro-survival or pro-death role in different physiological and pathological conditions [28, 29]. Autophagy has been shown to protect cells from death under conditions of starvation, growth factor withdrawal and neurodegeneration, but is also a critical contributing factor in certain types of cell death [30, 31]. In this study, we found that autophagy promoted TNF-α plus IFN-γ-induced apoptosis of MSCs, suggesting that autophagy performed different functions under inflammation and starvation conditions. Thus, it is feasible to modify autophagy in MSCs to achieve optimal therapeutic efficacy.

It is generally accepted that ROS induces autophagy, and autophagy, in return, serves to reduce oxidative products [32, 33]. ROS are small and highly reactive molecules, including oxygen anions and free radicals, which can oxidize proteins, lipids and DNA. When tightly controlled, ROS participate in vital cellular signaling by modulating the activity of the oxidized targets [34]. They can directly or indirectly interact with critical signaling molecules to initiate signaling in a broad variety of cellular processes, such as nuclear factor kappa B, MAP kinases, PI3 kinase and protein tyrosine phosphatases signal pathways [35]. Activation of these signal pathways has also been shown to modulate gene expression, inflammatory responses and cell survival [36]. ROS mediate cell death through caspase-dependent apoptosis or necrosis when excessive ROS inactivate caspases through oxidation [32]. ROS can also promote cell survival through activation of pro-survival transcription factors such as nuclear factor kappa B, ERK and heatshock factors to increase expression of anti-apoptotic proteins such as Bcl-2 and Bcl-xl [23, 37, 38]. In the previous study, we found that generation of ROS was required for activation of ERK, as inhibition of ROS generation suppressed ERK activation resulting from inhibition of autophagy and inflammatory stimuli [14]. In this study, we demonstrated that autophagy suppressed the expression of Bcl-2 through inhibition of ROS generation and ERK activation. Blockade of ROS generation and ERK activation enhanced MSC apoptosis. Collectively, these data indicate that inflammatory cytokine-induced autophagy plays a pro-apoptotic role in MSCs and it does not benefit the survival of MSCs in inflammatory conditions. Therefore, inhibition of autophagy could be a possible approach to improve the survival of MSCs when they are transplanted for inflammatory diseases.

## Conclusions

In summary, our findings indicate that inflammatory condition-induced autophagy promotes apoptosis of MSCs in sepsis. Therefore, modulation of autophagy in MSCs may provide a novel approach to improve MSC-mediated therapy. And it will be valuable to investigate whether inhibition of autophagy may improve the survival of MSCs in other inflammatory diseases such as graft-versus-host disease and multiple sclerosis.

## Abbreviations

3-Ma: 3-Methyladenine
CLP: Cecal ligation and puncture
EAE: Experimental autoimmune encephalomyelitis
ELISA: Enzyme-linked immunosorbent assay
ERK: Mitogen-activated protein kinase 1/3
FBS: Fetal bovine serum
IFN: Ingterferon
IL: Interleukin
L-DMEM: Dulbecco’s modified Eagle's medium low glucose
MHC: Major histocompatibility complex
MSC: Mesenchymal stem cell
NAC: N-acetyl cysteine
NC: Negative control
PBS: Phosphate-buffered saline
PCR: Polymerase chain reaction
PGE2: Prostaglandin E2
PI: Prodium iodide
ROS: Reactive oxygen species
sh: Small hairpin
TNF: Tumor necrosis factor
